# New Trends in Fluorescent Nanomaterials-Based Bio/Chemical Sensors for Neurohormones Detection—A Review

**DOI:** 10.1021/acsomega.2c04134

**Published:** 2022-09-15

**Authors:** Kinga Halicka, Francesca Meloni, Mateusz Czok, Kamila Spychalska, Sylwia Baluta, Karol Malecha, Maria I. Pilo, Joanna Cabaj

**Affiliations:** ^†^Faculty of Chemistry and ^§^Faculty of Microsystem Electronics and Photonics, Wroclaw University of Science and Technology, Wybrzeze Wyspianskiego 27, 50-370 Wroclaw, Poland; ‡ Department of Chemistry and Pharmacy, University of Sassari, Via Vienna 2, 07100 Sassari, Italy

## Abstract

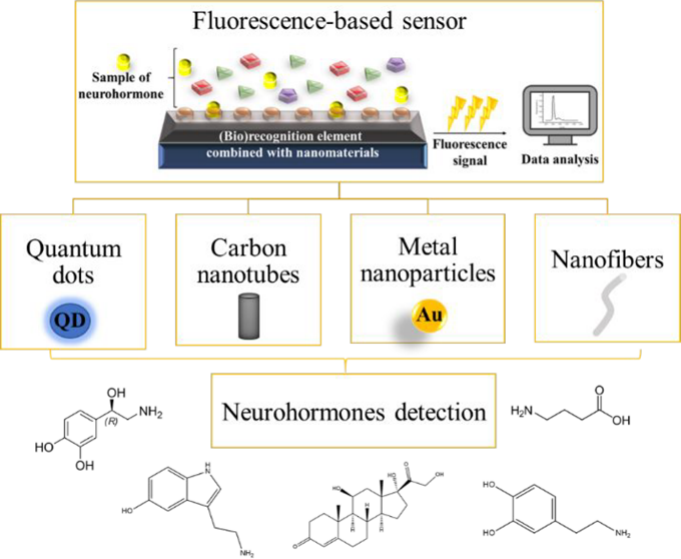

The study of neurotransmitters and stress hormones allows the determination of indicators of the current stress load in the body. These species also create a proper strategy of stress protection. Nowadays, stress is a general factor that affects the population, and it may cause a wide range of serious disorders. Abnormalities in the level of neurohormones, caused by chronic psychological stress, can occur in, for instance, corporate employees, health care workers, shift workers, policemen, or firefighters. Here we present a new nanomaterials-based sensors technology development for the determination of neurohormones. We focus on fluorescent sensors/biosensors that utilize nanomaterials, such as quantum dots or carbon nanomaterials. Nanomaterials, owing to their diversity in size and shape, have been attracting increasing attention in sensing or bioimaging. They possess unique properties, such as fluorescent, electronic, or photoluminescent features. In this Review, we summarize new trends in adopting nanomaterials for applications in fluorescent sensors for neurohormone monitoring.

## 1 Introduction

Nowadays, stress is a general factor affecting the population, and it may cause a wide range of serious disorders. Chronic stress can have a large pathophysiological impact on neuroendocrine^1^ and hormonal functions.^2^ Continuous stressful conditions may cause not only a wide range of affective disorders or anxiety but also cardiological or neurological disorders.^3−7^ All living organisms respond to stress or stressful environmental changes in a number of different ways. Neurohormones, which are the main regulators of the stress response, are physiologically active substances produced by the nervous system.^8^ As very often the first symptoms of stress-induced diseases (including psychiatric disorders, such as post-traumatic stress disorder) are underestimated, the development of biosensors that would enable the monitoring of parameters related to exposure to stressful conditions is extremely important. Knowledge about the level of neurohormones is an essential factor in modern medicine; however, there is no available method for accurate, sensitive, fast, and direct analysis that would allow monitoring the concentration of these species.

The evolution of biosensors was driven by the need for faster and more versatile analytical methods for application in important areas including clinical diagnostics, food analysis, environmental monitoring, and industry analysis in complex sample matrices (blood, serum, urine, food), with minimal sample pretreatment. Nowadays, an increasing interest in combining nanotechnology with biosensor construction is observed.^9−12^ The employment of nanomaterials (NMs) in biosensors permits the use of many new signal transduction technologies in their manufacturing. Nanomaterials, e.g., nanoparticles (NPs), nanotubes, nanofibers (NFs), nanorods, or quantum dots (QDs), have large possible application in the construction of biosensors.^13^ These materials possess a wide range of different properties, and therefore they are included in functional materials (electronic, optical, and magnetic), which can be bound to the biological molecules and used in biosensors to determine or amplify different signals.^14^ The unique optical properties of NMs, especially quantum dots, make the NMs attractive fluorophores that can be used both in vitro and in vivo in various biological studies, where traditional fluorescent labels based on organic molecules do not provide long-term stability or high enough intensity or where a simultaneous detection of many signals is needed.^15^ In sensors, the signal detection is based on the registering of a change in one of the physical properties (optical, thermal, mechanical, magnetic, or electrical) of sensing materials induced by the interaction with the analyte. The main advantages of using the nanoparticles, e.g., changing their optical properties, such as emission color, intensity, polarization, or emission kinetics, can be used as the principle in optical sensors systems.^13^ The use of carbon- and cadmium-based nanomaterials for the construction of sensors requires adjusting their properties (their shape, size, color of emission, and position of the absorption band) adequately to the needs that arise. Moreover, to obtain specificity of NMs in their sensing action, the surface modification, called functionalization, must be applied first.^16,17^ There are several methods for detecting an analyte with NPs, e.g., emission quenching from NPs, increase in NPs’ emission due to passivation of the NPs’ surface by the analyte, and nonradiative Förster resonance energy transfer (FRET).^18^ Nanostructured materials with great potential for the easy identification of individual chemical compounds with minimized sample volumes and their treatment are particularly promising in the process of biosensors development. Such materials include, e.g., carbon nanotubes (CNTs), graphene, and quantum dots, which very often are combined in optical sensor/biosensor detection systems. Optical biosensors represent a common type of biosensors, where the detection is based on the interaction of the optical field with a biorecognition element.^19^ These tools are stable owing to their resistance to radio interference and other electromagnetic waves. It is worth mentioning that they usually show high sensitivity, while simultaneously measuring without causing contamination of the sample with the reaction product.^20^

**Figure 1:**
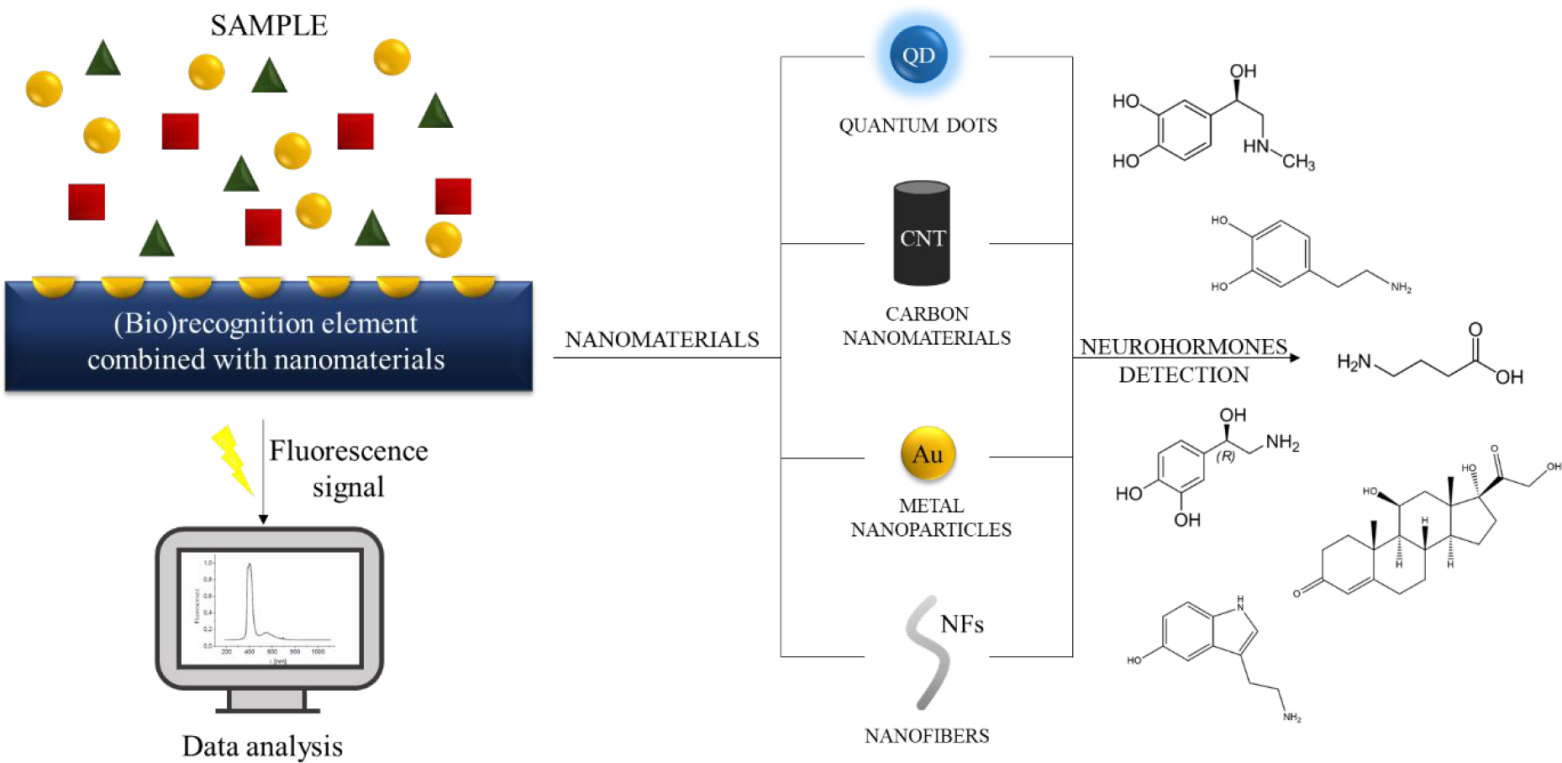
Schematic overview of fluorescent nanomaterial-based sensors for the detection of neurohormones.

Here, an overview of the recent developments in the field of nanomaterials-based optical sensors and biosensors that showed suitable detection limits to determine neurohormones in biological fluids is presented (Figure 1).

## 2 Operating Principles of Optical Sensors

Many various detection methods (electrical, electrochemical, piezoelectrical, thermal, etc.) can be utilized in the design of sensors and biosensors. One of the most accurate and at the same time versatile methods is the optical one. The operating principle of sensors using this method is based on several optical phenomena which can be divided into two groups–luminescent (fluorescence, chemiluminescence, electroluminescence, etc.) and nonluminescent (absorption, Raman spectroscopy, surface plasmon resonance, etc.).^21,22^ The most commonly used detection methods in optical sensors are based on fluorescence and absorption phenomena.

### 2.1 Absorption

Absorption is a phenomenon in which the photon energy of incident electromagnetic radiation is absorbed partially or in total by the examined analyte. As a result, the valence electrons of the atom are excited to higher energy levels, and subsequently absorbed energy may be transformed into heat or emitted as other electromagnetic radiation (Yasuda, 2015).^23^ The quantity describing the ability of a substance to absorb light of a specific wavelength is absorbance. According to the well-known Lambert–Beer’s law, it is possible to determine analyte concentration based on absorbance measurements. The absorbance magnitude (*A*) can be expressed as the logarithm of the ratio of the incident light intensity (*I*_0_) and the light transmitted to the detector (*I*).^24,25^
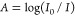
1
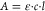
2where ε is the molar attenuation coefficient of the analyte (in cm^–1^ M^–1^), *l* is the optical path length (in cm), and *c* is the concentration of the analyte (in M).

In absorption spectroscopy, a light source of a given wavelength or selected spectrum is positioned opposite to a sensor. The key element in absorbance measurements is the selection of an optical path length that will provide an adequate signal-to-noise ratio. Depending on the design of the sensor and the nature of the performed measurements, the optical path may follow either perpendicular or parallel to the fluidic channel.^26^ Its length may be extended by incorporating, e.g., optical mirrors.^27^

### 2.2 Fluorescence

The phenomenon in which electromagnetic radiation is emitted as a result of exposure to radiation of a different, shorter wavelength is called fluorescence. A fluorophore or a fluorescent dye absorbs the energy of incident photons and is excited to a higher energy level as a result of radiation-free transitions. Over a short period of time, called the excited state lifetime, a vibrational relaxation of the fluorophore occurs. As a result, its energy is decreased, and eventually the electrons return to their valence band radiating photons.^28^ This radiation has a longer wavelength than the excitation one. The photon energy (*E*) is expressed as the product of Planck’s constant (*h*) and the frequency of the wave (ν), which can be presented as the ratio of the speed of light (*c*) and the wavelength (λ).
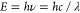
3

The wavelength difference between the excitation (λ_ex_) and emission (λ_em_) radiation is known as the Stokes shift and can range from 10 to 150 nm. The fluorophore can be excited numerous times, and the fluorescent emission can be obtained repeatedly before it is no longer able to fluoresce. This process is also known as photobleaching.^29^ The efficiency of the fluorescence process is determined by the quantum yield of the fluorophore and is a ratio of the emitted to the absorbed number of photons.^23^ Most commonly, fluorescence measurements are performed utilizing radiation in the range from 250 to 750 nm.^28^ Selected common fluorescent labels along with their excitation and emission wavelengths are presented in Table 1. The fluorescence intensity and properties of the sensor can be significantly enhanced with the use of quantum dots.^13,30^

**Table 1:** Selected Fluorescent Probes and Dyes^a^

fluorophore	λ_ex_ (nm)	λ_em_ (nm)
CF350	347	448
DAPI dihydrochloride^b^	364	454
CF405S	404	428
fluorescein 5(6)-isothiocyanate	492	518
ethidium bromide^1^	518	608
rhodamine 6G	528	551
cyanine 3	550	570
Texas Red hydrazine	580	604
cyanine 5	649	670

a Fluorescent Probes, Labels, Particles and Stains. Sigma-Aldrich, Inc.;^32^ Tully and O’Kennedy, 2015.^31^

b Bonded to DNA.

Measurements based on the fluorescence phenomenon require an appropriate combination of the spectral properties of the fluorophore, the light source, and the photodetector. The spectral maximum of the excitation light intensity should be as close as possible to the wavelength of the absorption maximum of the fluorophore. Furthermore, the spectral sensitivity of the detector should be close to the spectral maximum of the emitted radiation and favorably immune to or separated from the excitation light. Any mismatch results in decreased fluorescence intensity and detector sensitivity.

### 2.3 Förster Resonance Energy Transfer

A phenomenon that is related to fluorescence is the Förster resonance energy transfer. This process is a nonradiative transfer of energy between a donor and an acceptor. The FRET phenomenon can occur when the emission spectrum of the donor partially overlaps with the absorption spectrum of the acceptor. After excitation, electrons of the donor fluorophore shift from the ground state to a higher energy level. In contrast to the fluorescence phenomenon, the donor does not emit photons. Instead, the energy is transferred to the acceptor, excites its electrons, and triggers the emission of radiation while returning to the ground state.^33^

Moreover, FRET requires that the dipole moments of the donor and acceptor are properly aligned as well as that the distance between them is lower than 10 nm. This is because of the significant influence of the distance on the energy transfer capability.^34^ The quantum efficiency of the energy transfer is given by the Förster formula:
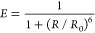
4where *E* is the FRET efficiency, *R* is the distance between the donor and acceptor, and *R*_0_ is the distance at which the efficiency of the energy transfer is 50%. The highest efficiency of the energy transfer is obtained in the donor–acceptor distance range from 0.5 to 1.5 of the *R*_0_ distance. Thus, this phenomenon can be used as a molecular ruler to measure the distance between molecules.^35^

## 3 Structure of Sensors and Biosensors

Sensors and biosensors that utilize optical detection methods require several elements to be considered during development, such as the source of the excitation light, the optical filters (e.g., to isolate excitation light from emitted light), and the photodetector that records the light intensity and produces a measurable value.^36^ Additional components like optical fibers, mirrors, couplers, and even lenses should also be considered.

**Figure 2:**
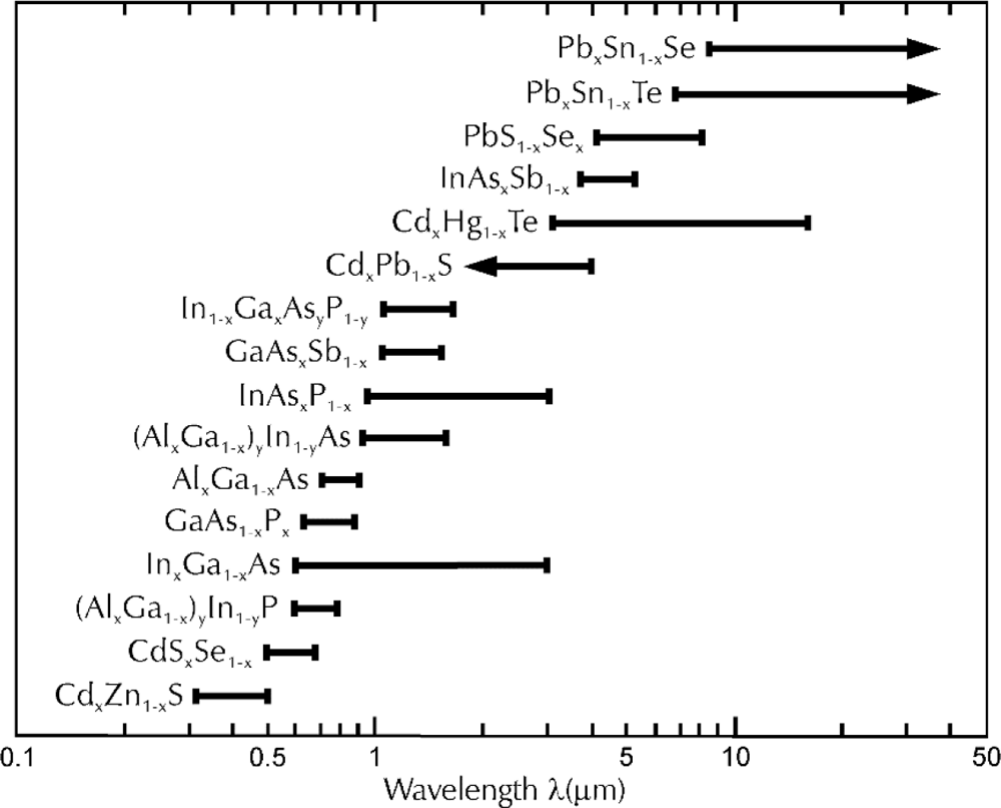
Emission wavelengths for different semiconductor compounds.

Novel optical-based biosensors utilize relatively small-sized light sources that are based on semiconductor compounds (mainly from the III-V and II-VI groups). The most popular light sources are semiconductor lasers, superluminescence diodes, and light-emitting diodes (LEDs). Light emissions for different semiconductor compounds that are used for the fabrication of light sources are presented in Figure 2.

As a photodetector, mainly a photodiode, phototransistor, or charge-coupled device (CCD) is used. In addition, CMOS-type (complementary metal oxide semiconductor) detectors (e.g., TCS3414, ams AG) and mini-spectrometers (e.g., C12880MA, Hamamatsu) have increasingly been used in sensing applications in recent years.^37,38^ The base materials used to fabricate these devices are, e.g., silicon, germanium, indium gallium arsenide, and lead sulfide. The wavelength ranges of exemplary semiconductors and semiconductor compounds that are used for photodetectors are presented in Table 2.

**Table 2:** Selected Materials Used for Photodetectors

material	λ (nm)
silicon, Si	190–1100
germanium, Ge	400–1700
indium gallium arsenide, In_*x*_Ga_1–*x*_As	800–2600
lead sulfide, PbS	<1000–3500

**Figure 3:**
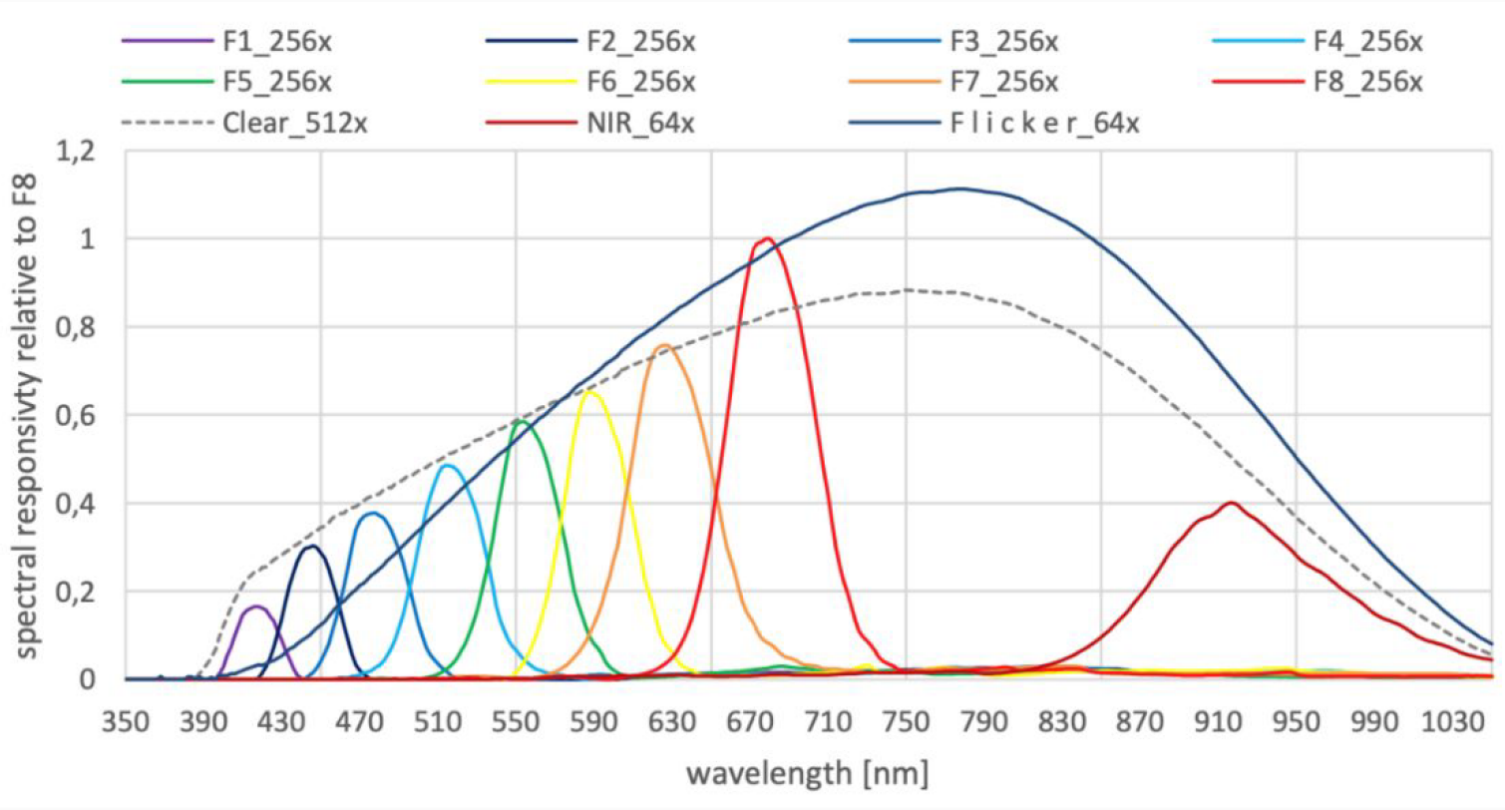
Spectral responsivity of the AS7341 11-channel multispectral digital sensor (AS7341 (ams AG)). Fx—channel number from the VIS spectrum; Fx_256x—channel gain value. Courtesy of ams AG, 2021.^39^

As can be seen in Figure 2 and Table 2, the semiconductive materials applied for both the light source and the photodetector cover the analytically useful range from UV to NIR and can be successfully used in the fabrication of miniature optical-based biosensors. Some of the new photodetectors on the market have built-in optical filters, making it possible to construct new, versatile, and relatively inexpensive miniature biosensors capable of measuring many different analytes in one single device. An example of such a detector is the AS7341 from ams AG. It is an 11-channel multispectral sensor with 8 channels covering the visible spectrum with integrated high-precision optical filters on a standard CMOS silicon structure (AS7341 (ams AG)). Each channel can be configured independently, including its integration time and gain. The spectral responsivity of the AS7341 sensor is presented in Figure 3.

**Figure 4:**
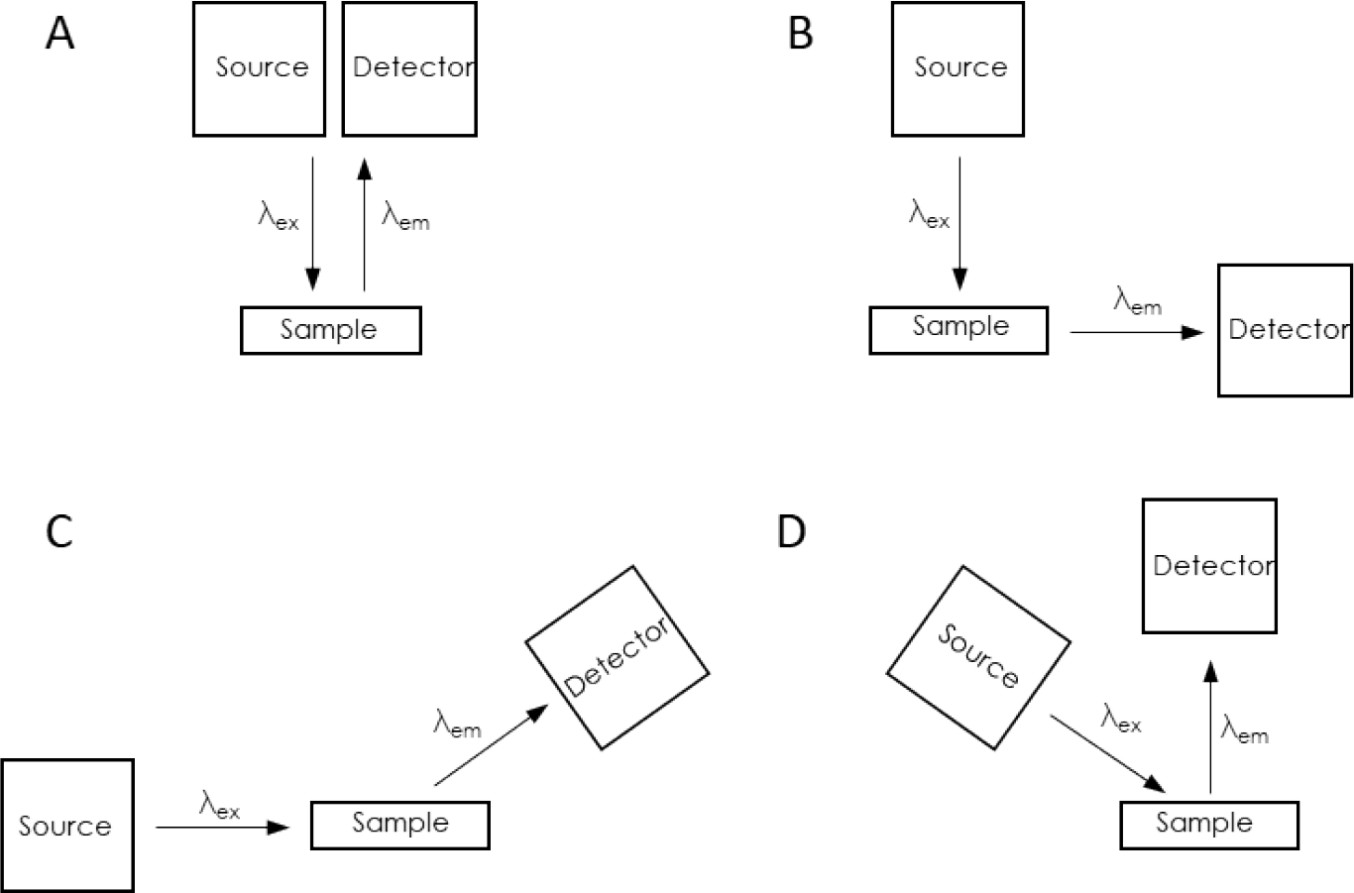
Measurement system configurations for fluorescence spectroscopy. (A) epifluorescence; (B) perpendicular configuration; (C and D) angular configuration.

In fluorescence spectroscopy, we observe the light emitted by the tested object; therefore, the light source should be positioned perpendicular to the photodetector or be separated by a set of mirrors and filters. Possible configurations of the light source with respect to the photodetector are shown in Figure 4. The perpendicular and angular configurations are used to separate and reduce the level of the background (excitation) signal from the emitted fluorescent signal measured by the photodetector. The most commonly used optical configuration in fluorescence spectroscopy is one in which the source and photodetector are faced perpendicular to each other.

## 4 Nanomaterials in the Construction of Fluorescent Sensors

Nanomaterials are promising structures for applications in sensors. A nanomaterial, as defined by ISO standards, is “*a material with any external dimension in the nanoscale (length range approximately from 1 to 100 nm) or having internal structure or surface structure in the nanoscale*”.^40^ Nanomaterials possess specific chemical, physical, and biological properties.^41^ They are characterized by large surface area to volume ratio, ease of functionalization, porosity, and therefore high loading capacity, and in some cases unique optical properties, thanks to which they find applications in the construction of optical sensors and biosensors, enabling their miniaturization and improving their performance, limit of detection, and response time.^42,43^ Functionalization of nanomaterials may additionally enhance the binding affinity toward the target, improve the stability in aqueous solutions, and give desired properties.^44^

A variety of nanomaterials have found application in the construction of fluorescent sensors (Table 3). The choice of nanomaterial can dictate the size, the possibility of modification and biomolecule immobilization, the detection technique used, or the bioapplicability of the sensing platform, so careful consideration of the NMs is encouraged. For instance, quantum dots, although relatively easily synthesized and exhibiting high quantum yields, can be toxic and require proper functionalization for aqueous solubility; gold nanoparticles (AuNPs) are biocompatible but are limited by aggregation and low stability after functionalization; silica NPs do not exhibit self-fluorescence; and biocompatible carbon and graphene quantum dots require excitation mostly in the UV range. Moreover, it is noteworthy that the addition of a biomolecule as a recognition element, for example, an enzyme, an antibody, or an aptamer, can further improve the sensitivity and selectivity of the constructed detection platform, which is why it is important to take the possibility of biomolecule functionalization under advisement when designing sensors.^45^

**Table 3:** Fluorescent Sensors Based on Various Nanomaterials for the Detection of Neurotransmitters^a^

nanomaterial type	sensor	analyte	linear range	LOD	ref
quantum dots	Ab–CdSe/ZnS-QDs	cortisol		100 pM	(46)
	aptamer–CdSe/ZnS-QDs		0.4–400 nM	1 nM	
	l-cys-capped InP/ZnS-QDs	DA	800 pM–100 nM	875 pM	(47)
	CdTe-QDs	NE	0.005–10 μM	2.1 nM	(48)
	TGA–CdS-QDs	DA	46.7 nM–0.394 μM	2.55 nM	(49)
	QDs@SiO_2_@MIPs	5-HT	0.28–2.8 μM	3.91 nM	(50)
	CdTe@SiO_2_@MIP	NE	0.04–10 μM	8 nM	(51)
	CdTe@SiO_2_ and CdTe/CdS/ZnS/SiO_2_	NE	0.08–20 μM	9 nM	(52)
	APTES-capped ZnO-QDs	DA	0.05–10 μM	12 nM	(53)
	aptamer–Ru complex-QDs	DA	0.03–0.21 μM	19 nM	(54)
	CdSe/ZnS-QDs	DA	100 nM–20 μM	29.3 nM	(55)
	F-CuInS_2_-QDs	DA	0.5–40 μM	0.2 μM	(10)
	CuInS_2_-QDs	EP	3 × 10^–5^–5 × 10^–7^ M	0.2 μM	(56)
	CdTe-QDs@silica	DA	0.5 μM–0.1 mM	0.241 μM	(57)
metal NPs	ds-DNA-templated Cu-NPs	DA	0.0001–10 μM	20 pM	(58)
	Au nanoflowers/Tb^3+^	DA	0.8–300 nM	0.21 nM	(59)
	Tb^3+^/AgNPs	DA	2.4–140 nM	0.42 nM	(60)
	ZnSa NW–AgNPs	DA	0–300 nM	3 nM	(61)
	Cu NPs	EP	1 × 10^–8^–1 × 10^–4^ M	3.6 nM	(62)
	Tf–Au-NCs	5-HT	0.2–50 μM	49 nM	(63)
	AgNPs	NE	8.92 × 10^–3^–5.66 × 10^–5^ M	5.59 μM	(64)
carbon-based NMs	B–N-CDs	DA	1 pM–1 μM	0.1 pM	(65)
	CNDs	DA	0–20 μM	47 pM	(66)
	DNA-SWCNTs	EP		0.5 nM	(67)
	CDs@MIP	DA	25–500 nM	1.7 nM	(68)
	N-doped carbon NPs	EP	0.1–50 μM	88 nM	(69)
		NE	0.1–50 μM	91 nM	
		DA	0.5–50 μM	140 nM	
	FAM-DNA/SWCNHs	DA	0.02–2.2 mM	5 μM	(70)
	CDs/ABPA/NADP^+^	GABA	0–90 μM	6.46 μM	(71)
	CDs	DA	33–1250 μM	33 μM	(72)
graphene-based NMs	PPy -GQDs	DA	5–8000 nM	10 pM	(73)
	GQDs	DA	0–60 μM	8 nM	(9)
	GQDs	DA	1–40 μM	22 nM	(74)
	multifarenes[3,3] hybridized with rGO	5-HT		55 nM	(75)
	lac-polymer-GQDs	DA	1–200 μM	80 nM	(76)
	GQDs	DA	0.25–50 μM	90 nM	(77)

a DA, dopamine; NE, norepinephrine; 5-HT, serotonin; EP, epinephrine; GABA, gamma-aminobutyric acid; NPs, nanoparticles; Ab, antibody; QDs, quantum dots; TGA, thioglycolic acid; MIP, molecularly imprnted polymer; APTES, (3-aminopropyl)triethoxysilane; F-CuInS_2_ , 3-aminophenyl boronic acid-functionalized CuInS_2_; ZnSa NW, zinc-salophen nanowire; Tf, transferrin; NCs, nanoclusters ; B–N-CDs, bifunctionalized carbon dots with boronic acid and amino groups; CNDs, sulfur-doped carbon dots; CDs, carbon dots; SWCNTs, single-walled carbon nanotubes; FAM, 5-carboxyfluorescein; SWCNHs, single-walled carbon nanohorns; ABPA, 3-aminophenylboronic acid; NADP^+^, nicotinamide adenine dinucleotide phosphate; PPy, polypyrrole; GQDs, graphene quantum dots; rGO, reduced graphene oxide; lac, laccase.

### 4.1 Carbon Nanotubes

According to ISO Standards, a carbon nanotube is a “*hollow nanofiber composed of carbon*”.^78,79^ The tubular shape of CNTs comes from curled-up graphene sheets. Depending on the number of cylindrical graphene layers, CNTs can be classified into single-walled CNTs (SWCNTs, a single one-atom-thick graphene sheet) or multiwalled CNTs (MWCNTs, several SWCNT layers). The former attracted special attention in sensing and biosensing research. SWCNTs can have a zigzag, armchair, or chiral structure, which are then classified as semiconducting, metallic, or semimetallic, respectively. The most advantageous properties of these one-dimensional structures include, apart from large surface area and mechanical properties, electronic, electrochemical, and photophysical properties, electrochemical stability, and thermal and electrical conductivity. In comparison to other methods used in biosensors, such as antigen–antibody interactions, CNTs present a stable, low-cost, reproducible material that does not require living organisms for production. In addition, their nanoscale size may lead to precise targeting of the molecules.^80−82^

The physical construction of SWCNTs defines their optical properties. Because of their nanoscale size (ranging from 0.7 to 3 nm in diameter), they are subject to the quantum confinement effect, which leads to near-infrared fluorescence in the range of 900–1600 nm. This fluorescence is sensitive to its local environment and depends on several factors, such as the diameter and chiral vectors of the CNT, the local dielectric environment, charge transfer, and the presence of fluorophores or Coulombic interactions. SWCNTs exhibit strong resonance Raman scattering and possess a broad absorption spectrum, which makes it possible to use them as quenchers for different fluorophores.^80−84^

Initially, SWCNTs are hydrophobic and tend to aggregate. Properly selected surface modification, covalent or noncovalent, can make them not only hydrophilic but also biocompatible, adapting them for applications in sensors and biosensors. It also tunes the fluorescence properties of SWCNTs. Properties, including high photostability, lack of photobleaching and lack of blinking, various chiralities, and biomolecule comparable dimensions, make SWCNTs a good material for sensing applications.^81,84^ Functionalization creates an organic phase, called a corona, on the surface of CNTs. A perfect coating should be nontoxic, biocompatible, and stable and possess specific functional groups that allow further modification; for example, modification with oxygenated moieties may leave carboxylate groups on the surface of CNTs. These groups improve water solubility and can be used for further bioconjugation. Dispersion in aqueous solutions is also possible by using surfactants, such as sodium dodecyl sulfate, or soluble molecules, including proteins, nucleic acids, and polysaccharides. In the case of covalent modification, oxidation and cycloaddition are often used.^67,80,84^

SWCNTs have been applied in optical sensors for the detection of, e.g., biomarkers (e.g., toward cancer^83^ or glucose levels^85^), proteins,^81^ metals,^86^ or neurotransmitters.^67^

### 4.2 Graphene Quantum Dots

Carbon nanotubes are not the only carbon nanomaterial that has found application in optical sensors. Carbon dots (CDs) are another promising carbon-based NM. CDs are nearly spherical, fluorescent carbon materials with one dimension less than 10 nm. Their synthesis is cheap and biofriendly, and they are biocompatible, soluble, and resistant to photobleaching. Because of abundant functional groups, such as hydroxyl, carboxyl, and amine, they can be easily functionalized. They possess high fluorescence stability and strong absorption in the ultraviolet region, expandable to the visible region, they do not blink, and the excitation and emission wavelengths are adjustable. CDs-based sensors have been developed for the detection of, for example, metal ions, pesticides, and biomolecules such as nucleic acids or for use in food safety.^87−89^

Graphene quantum dots (GQDs) are zero-dimensional, fluorescent nanomaterials consisting of one or few layers of graphene. As a carbon-based material, GQDs present excellent biocompatibility and low to no toxicity, as well as good solubility in various solvents, including aqueous solutions. They possess properties characteristic of both graphene and quantum dots. They exhibit good mechanical, electrical, thermal, and optical properties, as well as large surface area, chemical inertness, high fluorescence activity, and photoluminescence stability.^90−94^ In addition, because of their fluorescent properties, granted by the radiative recombination of electron–hole pairs, they can serve as fluorescent labels or quenchers. The nanoscale allows for the quantum effects, exciton confinement, and quantum confinement leading to a band gap and edge effects, which lead to optical and electrical properties unobtainable in classic quantum dots. GQDs can be easily functionalized, as a large number of sites for potential modification is provided by their structure. Functionalization can be carried out using organic molecules and biomolecules, such as proteins, amines, nucleic acids, or antibodies, or using nanomaterials, creating hybrid NMs.^90,91,94,95^

There are two main categories of synthesis of GQDs: the top-down approach and the bottom-up approach. The first one is a simpler method that involves a fragmentation of carbon materials, such as graphene oxide, CNTs, graphite, or carbon fibers, through, e.g., chemical or electrochemical oxidation. However, control of the size and morphology of created GQDs is not possible. The second approach involves several chemical reactions from smaller precursors, such as citric acid or polycyclic aromatic hydrocarbon, to create GQDs, e.g., hydrothermal treatment and chemical vapor deposition. Examples of methods for the synthesis of GQDs are presented in Table 4.^90,93^ Optical sensors based on GQDs have been used to detect, for instance, metal ions (such as copper(II),^94^ iron(III),^96^ and mercury(II)^97^) or organic compounds (e.g., pyrene,^95^ propofol,^98^ and glucose^99^).

**Table 4:** Examples of Synthesis Methods of Graphene Quantum Dots

top-down methods	bottom-up methods
chemical oxidation	hydrothermal treatment
electrochemical oxidation	pyrolysis
hydrothermal treatment	thermolysis
solvothermal treatment	carbonization
microwave-assisted cutting	chemical vapor deposition
ultrasonication	precursor reduction
acid vapor cutting	intramolecular condensation

### 4.3 Quantum Dots

Quantum dots are defined as “*crystalline nanoparticles that exhibit size-dependent properties due to quantum confinement effects on the electronic states*”.^100^ They are semiconducting, inorganic, fluorescent nanocrystals, usually synthesized from elements of groups II-VI or III-V. Their size is limited by the exciton Bohr radius of the material and usually ranges from 1 to 10 nm. Special focus is placed on QDs with the size of 2–6 nm, as they resemble biomolecules.^101−104^ Optical properties, which can be tuned by controlling the size of QDs, are characterized by narrow emission spectra, broad excitation spectra, long fluorescence decay lifetime, high quantum yield, and lack of photobleaching.^102,103,105^ QDs can have a core–shell-like structure, where the core is surrounded by another layer, usually made of a different compound. The shell protects the core from photo-oxidation and improves the quantum yield.^106^

One of the most popular methods of synthesis of QDs is the hot-injection method. In this technique, precursors of the core compounds are injected into a hot surfactant, starting the process of nucleation. Then the temperature is lowered to let the crystals grow, and the process is stopped when the nanocrystals reach the desired size. The surfactant molecules become the ligands on the surface of the NPs.^105^ Green synthesis has also been described, where toxic compounds are replaced with environmentally friendly substances and lower temperatures can be used.^107^

**Figure 5:**
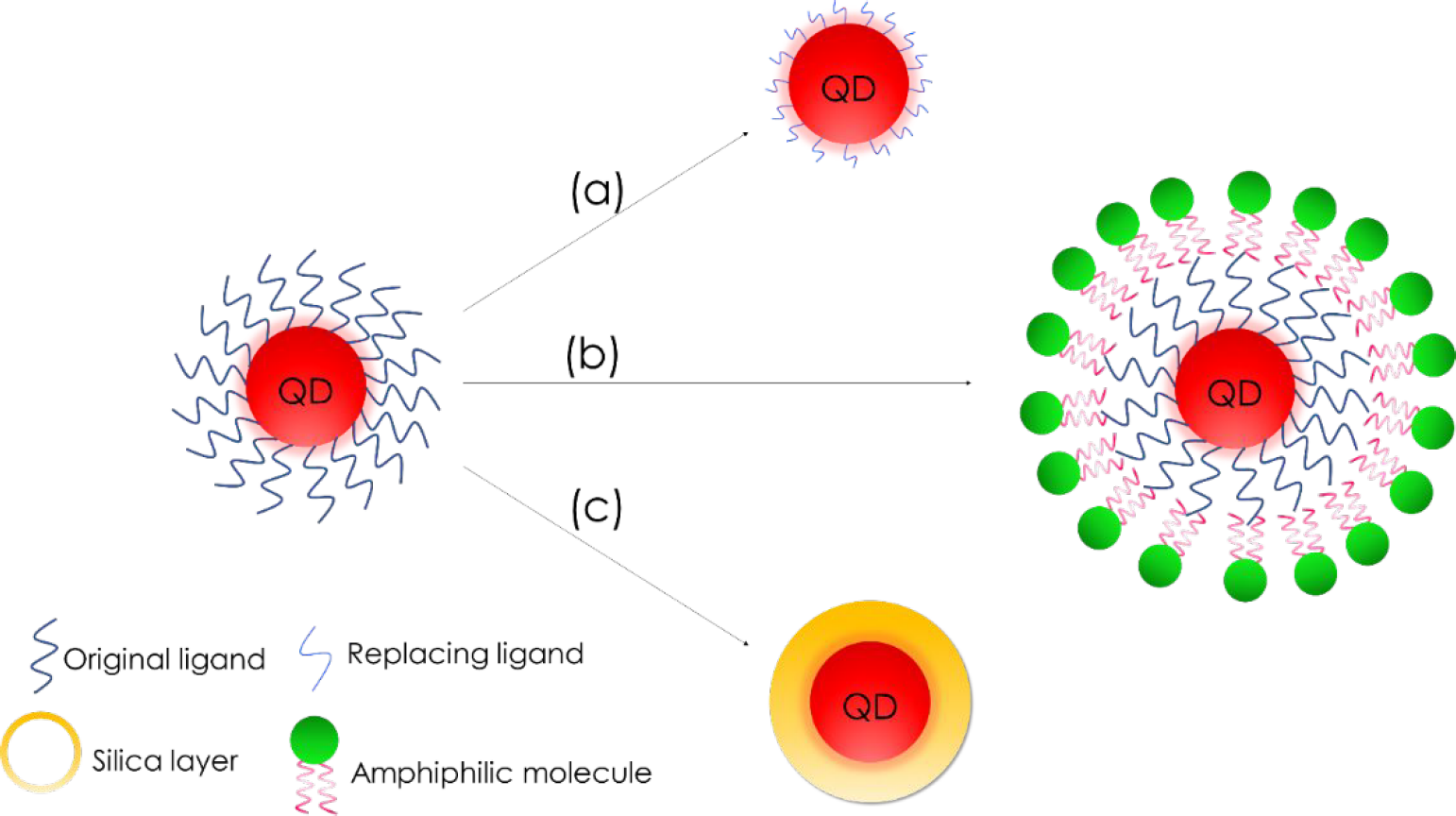
Surface modification of QDs: (a) ligand exchange method, (b) amphiphilic molecules coating, and (c) silanization.

The organometallic synthesis of QDs causes the requirement of surface modification in order to obtain hydrophilic QDs that could be further functionalized and applied in biomedicine and sensing. Most commonly used techniques include ligand exchange, silanization, and amphiphilic coating (Figure 5).^108^ The ligands on the surface of QDs affect their size, shape, optical and physicochemical properties, colloidal stability, and dispersion in polar and nonpolar environments, as well as further bioconjugations.^108,109^ The simplest modification method is ligand exchange, where hydrophobic surface molecules are replaced with molecules with two functional groups, hydrophobic for the surface binding (often a thiol group, −SH) and hydrophilic (e.g., carboxyl group, −COOH) for water solubility. Replacement ligands may include, e.g., 3-mercaptopropionic acid or d-penicillamine.^110−112^ Silanization is the synthesis of a siloxane layer around a single quantum dot, and amphiphilic molecules can create a cross-linked polymer layer around a single QD.^108,110^

Because of their unique optical properties, QDs have found application in many optical sensors,^13^ for instance, in the detection of metal ions,^113^ pesticides^114^ or toxins,^115^ sugars,^116^ microorganisms,^117^ and biomolecules, such as proteins,^118^ nucleic acids,^119^ neurotransmitters,^120^ or vitamins.^121^

### 4.4 Gold Nanoparticles

Gold nanoparticles (AuNPs), also known as colloidal gold, are stable nanoparticles with a size between 1 and 100 nm. They possess unique optical, physicochemical, electronic, and catalytic properties that make them applicable in various fields. They are characterized by large surface area, distinct shape, stability, strong adsorption, absorption in the visible spectrum region (with a possible shift to the UV–visible region depending on the size and morphology), the possibility of functionalization, and optical properties dependent on the size. They present surface plasmon resonance, the ability for fluorescence quenching due to fluorescence resonance energy transfer, and surface-enhanced Raman scattering (SERS). To improve the properties, stability, dispersity, functionality, and biocompatibility, surface modification is required. Functionalization is possible through covalent bonding, electrostatic interactions, and molecules binding.^122,123^

Because of these features, AuNPs can be used in sensing and biosensing technologies, for instance, for the detection of metal ions,^124^ anionic contaminants,^122^ pesticides, and drugs.^123^

### 4.5 Nanofibers

Nanofibers are one-dimensional “*nano-objects with two external dimensions in the nanoscale and the third dimension significantly larger*”.^78^ Various morphologies of NFs can be obtained, depending on the process conditions, e.g., smooth, beaded, hollow, or core–shell structures.^125^ NFs are characterized by excellent mechanical, physical, and chemical properties, including a high surface area to volume ratio and porosity. In addition, they offer flexibility in design, easy and low-cost fabrication, and a wide range of functionalization molecules and approaches.^126,127^

Electrospinning is one of the simplest and therefore commonly used techniques for NFs fabrication. In short, after being electrified, a liquid droplet generates a jet, which is then stretched and elongated, creating fibrous structures. The basic setup includes a syringe filled with a solution of a chosen polymer along with a syringe pump, a spinneret (usually a needle), a conductive collector (plate or rotating), and a high-voltage power supply. Charging of the liquid forces the droplet to form a Taylor cone, and the jet is ejected. The jet, initially in a straight line, undergoes whipping motions and stretching, solidifies, and is then collected on the collector in the form of nanofibrous mats or membranes.^128^

**Figure 6:**
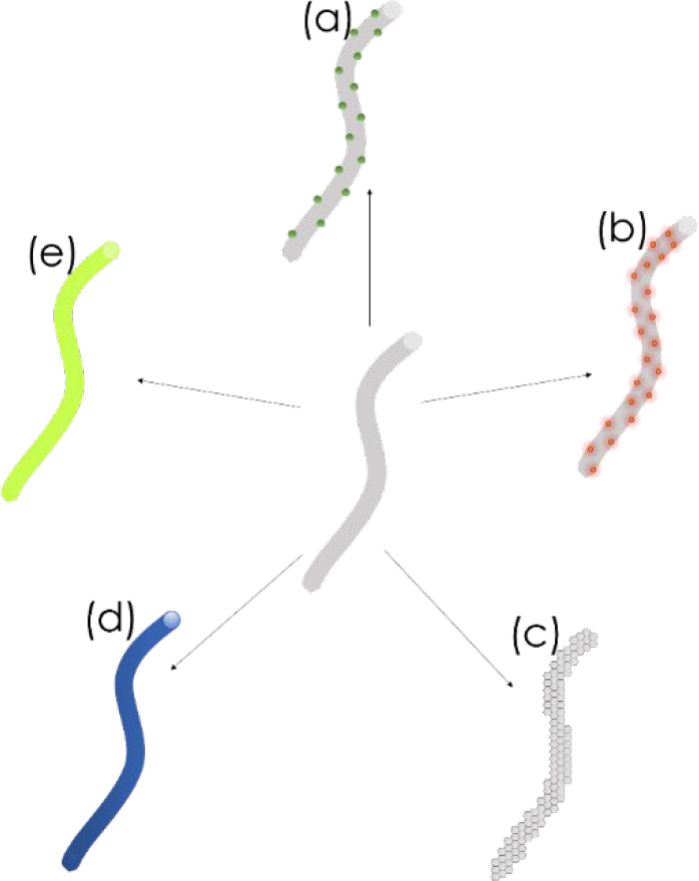
Modification of nanofibers with (a) metallic NPs, (b) semiconducting NPs, (c) carbon NMs, (d) conjugated polymers, and (e) organic dyes.

Functionalization of NFs can be achieved through a direct blending of functional materials (such as chromophores or NPs) with polymers, followed by electrospinning of the solution, or through surface modification of NFs by physical adsorption or suitable chemical reaction. The second approach is especially useful in sensing and biosensing, as the large surface area allows the immobiliation or encapsulation of multiple molecules, leading to improved sensing performance. Moreover, the use of NFs does not contaminate the solution, as they can be easily removed from the sample after the detection.^126,127^ The incorporation of carbon NMs, metallic NPs, semiconducting NPs, conjugated polymers, or organic dyes (e.g., graphene, gold nanoclusters, QDs, polyfluorene, and rhodamine derivatives, respectively) makes NFs a promising platform for sensing and biosensing (Figure 6).^127,129−133^

Nanofibers were applied in optical sensors for the detection of, e.g., heavy metals,^127^ environmental toxicants,^134^ cancer cells,^135^ and pH.^133^

## 5 Sensors and Biosensors Based on Nanomaterials in the Detection of Neurohormones

Concentrations of neurotransmitters produced by the nervous system and of hormones produced by glands are indicators of the body’s state, and they are related to stress conditions.^136,137^ Abnormal levels of these molecules, such as dopamine, epinephrine, serotonin, cortisol, etc. can be found in response to several conditions, such as emotional state (stress, euphoria, fear, anger, etc.) or in the presence of various diseases, from neurodegenerative and cardiac pathologies to mental disorders and tumors.^137−143^ Therefore, it is very important to determine the concentration of neurohormones in human biofluids (serum, plasma, saliva, sweat, urine, cerebral spinal fluid, and platelets) for disease prevention or diagnosis, in order to improve the quality of life and to minimize health risks.

Owing to the low physiological concentrations of these biomarkers, ultrasensitive methods to carry out quantitative analysis in biological samples are necessary. In recent years, numerous analytical strategies, including HPLC,^144,145^ HPLC-MS,^146,147^ electrochemical methods,^148−151^ and UV–vis spectroscopy,^152^ have been developed. Despite their selectivity and sensitivity, most of these methods have some non-negligible drawbacks, such as consumption of time, high costs, qualified personnel need, and sometimes the need for additional steps. In this regard, optical (bio)sensors have appeared as promising useful analytical alternatives, possessing a limit of detection in the range of nanomolar or less and high reproducibility and sensitivity, in addition to rapid response, easy analysis, and low costs. Considering these, fluorescence-based (bio)sensors that use fluorescent nanomaterials are, in general, very useful analytical tools thanks to their high sensitivity, biocompatibility, and photostability.^63,141^

### 5.1 Detection of Epinephrine

**Figure 7:**
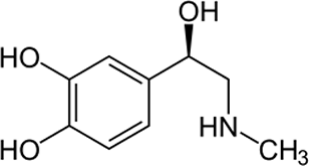
Chemical structure of adrenaline (epinephrine).

Adrenaline, also known as epinephrine (EP) (Figure 7), is one of the key neurohormones secreted by the adrenal medulla that transmits information in the central nervous system of mammals. Its role is significant in initiating glycogenolysis, increasing blood sugar levels, increasing lipolysis, and regulating the heart rhythm.^62,153^ In medicine, it is mainly used in the treatment of heart diseases, bronchial asthma, and anaphylactic shock.^56^ In free form, EP occurs as an organic cation in central nervous system tissues and body fluids.^154^ Many diseases are closely related to changes in adrenaline levels—for example, low levels of adrenaline have been found in patients with Parkinson’s disease.^62^ Hence, the quick and sensitive detection of EP in body fluids is of key importance in medical diagnostics, pharmacology, and the control of nerve physiology.^155^ Currently, many tests for the determination of EP concentrations are known, such as electrochemical methods,^156−158^ capillary electrophoresis,^159,160^ liquid chromatography,^161,162^ or chemiluminescence.^163,164^ Unfortunately, most of these methods are limited by high costs, tedious operation, and the need to prepare the sample for measurements in advance. In the case of EP, there are almost no existing optical detection systems. It is much more difficult to detect EP because of its rapid metabolism, and in fact, it does not possess any fluorescence properties.^155^

Z. Liu and S. Liu have developed a simple fluorescence biosensor for the detection of EP.^56^ For this purpose, water-soluble CuInS_2_ quantum dots closed with l*-*cysteine were synthesized. In the next stage, positively charged EP was accumulated on the surface of quantum dots through electrostatic interactions and hydrogen bonds, resulting in the formation of electrostatic adrenaline–CuInS_2_ QDs complexes. The constructed system also uses tyrosinase, which can stimulate EP to generate H_2_O_2_ and additionally oxidizes adrenaline into EP quinone. Both H_2_O_2_ and epinephrine quinone can suppress CuInS_2_ fluorescence through an electron transfer process. Under optimal conditions, the fluorescence -quenching coefficient was proportional to the logarithm of the EP concentration in the concentration range of 1 × 10^–8^–1 × 10^–4^ mol L^–1^, while the detection limit was 3.6 nM.

Another method of detecting EP was proposed by Baluta and co-workers.^153^ A miniature biosensor based on low-temperature cofiring ceramics technology (LTCC) was designed by the immobilization of enzymes belonging to the class of oxidoreductases (laccases, tyrosinases) on the surface of a semiconducting polymer (poly(2,6-di([2,2′-bithiophen]-5-yl)-4-(5-hexylthiophen-2-yl)pyridine)). The detection procedure was based on the oxidation of the substrate in the presence of the enzyme. An alternative enzyme-free system resulted in the formation of a colored complex between Fe^2+^ ions and EP molecules. Under optimized conditions, the sensor was characterized by very high sensitivity and selectivity over a wide range of concentrations with a detection limit of 0.14–2.10 nM. The constructed system was successfully used to determine EP in labeled pharmaceutical samples.

Sivasankaran and Girish Kumar proposed a new EP detection strategy based on colorimetric and fluorescence measurements resulting from the formation of copper nanoparticles from a CuCl_2_ solution.^62^ Visual detection was also possible by changing the color of the solution from pale blue to red-brown. In this case, the CuCl_2_ solution is used as a probe to simplify the method, and its performance is comparable to that of fluorometric and colorimetric sensors. The created sensor is characterized by simplicity, high selectivity, and repeatable operation. In fluorometric detection, it showed activity in the linear range of 3 × 10^–5^–5 × 10^–7^ M, while in colorimetric detection in the range of 5 × 10^–4^–2 × 10^–5^ M. To confirm the commercial applicability, artificial urine and pharmaceutical preparations were successfully analyzed by the constructed two-channel sensor.

Mann et al. used SWCNTs functionalized with DNA to create a fluorescent sensor for the detection of catecholamine neurotransmitters.^67^ They selected 10 different DNA sequences to modify the corona phase of CNTs and used near-infrared microscopy to measure the fluorescence of the obtained probes. In the case of epinephrine, the limit of detection (LOD) was in the range of 0.5–33.3 nM, depending on the oligonucleotide sequence used for the modification. The same experiment was repeated for dopamine and norepinephrine, with the LOD between 0.7 and 9438 nM and 0.5 and 23.7 nM, respectively. In addition, it was found that selected sensors were able to distinguish different neurotransmitters at low concentrations of 50 nM.

Highly crystalline nitrogen-doped fluorescent carbon nanoparticles (N-CNPs), synthesized from ethylene glycol and alanine anhydride, were developed by Das and Dutta^69^ and exhibited blue fluorescence. These NPs were applied for the detection of neurotransmitters, epinephrine, norepinephrine, and dopamine, via fluorescence recovery. In the proposed strategy, the system’s fluorescence intensity was quenched by MnO_4_^–^ and recovered with the help of the analytes. The detection limits were 88, 91, and 140 nM for EP, NE, and DA, respectively. Additionally, the system showed good selectivity toward potential interfering agents and good recovery values in real sample analysis.

The sensor systems for the detection of EP described above are summarized in Table 5.

**Table 5:** Optical Sensors for Epinephrine Determination

sensing platform	linear range	LOD	ref
CuInS_2_-QDs	3 × 10^–5^–5 × 10^–7^ M	0.2 μM	(56)
poly(2,6-di([2,2′-bithiophen]-5-yl)-4-(5-hexylthiophen-2-yl)pyridine	0.14–2.10 nM	0.14–2.10 nM	(153)
Cu-NPs	1 × 10^–8^–1 × 10^–4^ M	3.6 nM	(62)
DNA-SWCNTs		0.5 nM	(67)
N-doped carbon NPs	0.1–50 μM	88 nM	(69)

### 5.2 Detection of Norepinephrine

**Figure 8:**
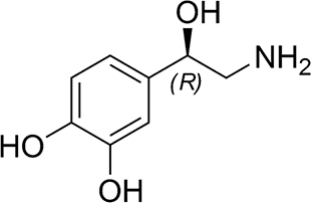
Chemical structure of noradrenaline (norepinephrine).

Noradrenaline, also called norepinephrine (NE) (Figure 8), like EP belongs to the group of catecholamines and has a number of different functions, also acting as a stress hormone. One of the most important functions of NE is its role as a neurotransmitter that is released from sympathetic neurons and acts on cardiac function. As a stress hormone, it affects parts of the brain that control attention and response, such as the amygdala.^64^ The determination of NE concentration is extremely important in modern medical diagnostics, not only for the determination and study of the physiological processes of this catecholamine but also for the diagnosis and monitoring of the course of treatment of cardiovascular diseases and mental disorders.^165^ Moreover, overexpression of both NE and EP in blood and urine may indicate the presence of pheochromocytoma located in the adrenal medulla; however, the overexpression of only NE suggests the presence of a tumor elsewhere. Hence, tests enabling the simultaneous measurement of NE and EP concentration in blood and urine are extremely useful, as thanks to them it is possible to quickly diagnose the disease and implement appropriate treatment.^166^ Many methods for the detection of NE have been described, such as electrochemical detection,^167,168^ capillary electrophoresis,^169^ and HPLC-based methods.^146,170^ The downside to using these methods is the need for expensive equipment and manpower. Therefore, it is necessary to find fast and sensitive methods for the determination of this catecholamine in biological samples.

Menon et al. developed a two-channel colorimetric and fluorometric sensor for the sensitive and rapid detection of NE.^64^ The basis of the detection strategy is the formation of brown silver nanoparticles (AgNPs) in the presence of NE, resulting in a strong fluorescent signal. The designed sensor is characterized by a linear relationship between the absorbance values and the concentration of NE in the range 1.00 × 10^–6^ −6.66 × 10^–8^ M with a detection limit of 1.79 × 10^–8^ M. Additionally, it was found that the fluorescence intensity was directly proportional to the concentration of NE in the range 8.92 × 10^–3^–5.66 × 10^–5^ M with a LOD of 5.59 × 10^–6^ M. The system constructed in this way has been successfully used to determine NE in synthetic blood serum, which indicates its potential use for diagnostic purposes.

Zhang and his team proposed a different strategy. They constructed a ratiometric fluorescent nanoprobe consisting of water-soluble fluorescent carbon dots and 3-mercaptopropionic acid-coated cadmium telluride quantum dots (CdTe-QDs).^48^ During single-wavelength excitation, the hybrid nanoprobe generated double-emission peaks belonging to CDs and CdTe-QDs. The basis of the action was quenching of fluorescence by EP or NE due to electron transfer from QDs to the oxidation products of catecholamines. At the same time, the fluorescence intensity of the CDs remained unchanged. The relative ratios of the fluorescence intensity of CDs and CdTe-QDs were directly proportional to the concentration of catecholamines. The new fluorescence detection platform was fast and convenient and was used to detect EP and NE in human serum samples with satisfactory linear range results for NE from 0.005 to 10 μM and a LOD of 2.1 nM.

Wei et al. designed a molecular imprinted sensor based on CdTe@SiO_2_ quantum dots and molecularly imprinted polymer (MIP) for sensitive and fast detection of NE.^51^ The constructed matrix was characterized by Fourier transform infrared spectroscopy, transmission electron microscopy, and fluorescence spectroscopy. The newly synthesized system was characterized by high selectivity and affinity for NE. The basis of the sensor operation was the measurement of the fluorescence intensity of CdTe@SiO_2_@MIP in the presence of the tested catecholamine. The fluorescence intensity of the system decreased linearly with the increase of NE concentration in the range of 0.04–10 μM. The limit of quantification was set at 8 nM. The constructed MIP-based nanoplatform was successfully used in the analysis of NE concentration in the plasma of rats. Because of the simplicity and speed of the gun, it can potentially be used to determine NE in medical diagnostics.

Wei et al. also proposed another MIP-based method for the sensitive and rapid detection of NE and EP.^52^ For this purpose, they carried out a MIP-anchoring process on the surface of two different colored quantum dots. The platform made of CdTe@SiO_2_ and CdTe/CdS/ZnS/SiO_2_ QDs was modified with templates from NE and EP to obtain two matrices, NE-QD@MIP (for NE) and E-QD@MIP (for EP). Such constructed nanosensors were characterized by selectivity and high binding affinity to the appropriate matrix molecule. The matrix mixture could be excited at the same excitation wavelength, and simultaneous detection of NE and EP could be accomplished by monitoring two different color fluorescence signals without spectral overlap. Under optimal conditions, the fluorescence intensity of the system decreased linearly with increasing concentration of the standard molecule in the linear range of 0.08–20 μM with a detection limit of 9 nM for NE and 12 nM for EP.

Głowacz et al.^171^ developed a strategy based on excitation–emission fluorescence spectroscopy using glutathione-capped CdSeS/ZnS quantum dots (QDs-GSH) for the detection of various neurotransmitters, including EP, NE, GABA, and more, because of the cross-affinity of the modified nanocrystals toward different chemical structures of neurotransmitters. The proposed assay allowed the quantification of catecholamine neurotransmitters (epinephrine, norepinephrine, and dopamine) at the micromolar concentration range. However, in the case of other tested neurotransmitters (serotonin, GABA, and acetylcholine), the analysis of only specific compounds was possible, limiting the future use of this method. The authors state that further research regarding the LOD, selectivity, and real sample analysis must be done.

The sensor systems for the detection of norepinephrine described above are summarized in Table 6.

**Table 6:** Optical Sensors for Norepinephrine Determination

sensing platform	linear range	LOD	ref
AgNPs	8.92 × 10^–3^–5.66 × 10^–5^ M	5.59 × 10^–6^ M	(64)
CdTe-QDs	0.005–10 μM	2.1 nM	(48)
CdTe@SiO_2_@MIP	0.04–10 μM	8 nM	(51)
CdTe@SiO_2_ and CdTe/CdS/ZnS/SiO_2_	0.08–20 μM	9 nM	(52)
N-doped carbon NPs	0.1–50 μM	91 nM	(69)

### 5.3 Detection of Dopamine

**Figure 9:**
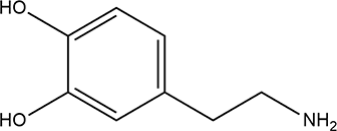
Chemical structure of dopamine.

Dopamine (DA) (Figure 9) is an endogenous neurotransmitter of the catecholamine family of which it is also the most abundant. It plays a key role in the functioning of the central nervous, renal, cardiovascular, and hormonal systems: it controls stress responses, consciousness, sleep–wake cycle, motivation, motions, memory formations, etc. High levels of DA can be observed in the presence of cardiotoxicity, which can lead to rapid heartbeat rate, hypertension, and also heart failure. On the other hand, low concentrations are typical of Parkinson’s disease, schizophrenia, Alzheimer’s disease, and depression.^140−143,179,180^

In agreement with the Human Metabolome Database, the physiological concentration of DA varies in different biofluids: in blood it is less than 130 pM, whereas in human cerebrospinal fluid and urine the levels of dopamine are ≈5 nM and less than 1 μmol/mmol of creatinine, respectively.^141,181,182^ As the concentration of DA in real samples is very low, its determination requires very sensitive methods.

**Figure 10:**
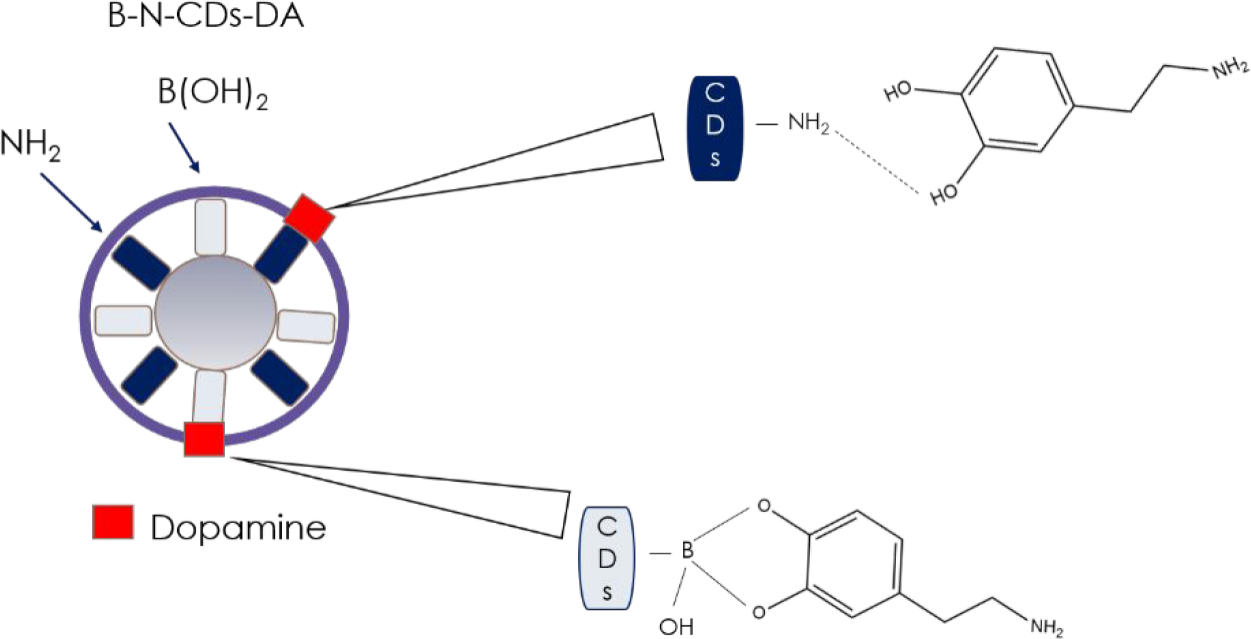
Detection of dopamine with B–N-CDs.

The best detection limit of 0.1 pM with a wide linear range (from 1 pM to 1 μM) was achieved by Liu and co-workers,^65^ who realized a fluorescent biosensor based on in situ bifunctionalized carbon dots with boronic acid and amino groups (B–N-CDs). 3-Aminophenylboronic acid was used as the unique precursor for modified CDs preparation using a simple hydrothermal approach. The DA-sensing process was based on the interactions between the amino groups on the CDs and DA, which enable the absorption of DA onto the surface of the CDs through the formation of hydrogen bonds and on those of the boronic acid group (B(OH)_2_) with the diol moiety of DA (Figure 10). Upon the addition of DA to B–N-CDs, the fluorescence intensity, measured at 418 nm, greatly enhanced, reaching an increase of 45% after the addition of 1 μM DA. The obtained biosensor was used for the determination of DA in human serum samples, giving recovery values between 93% and 106%.

**Figure 11:**
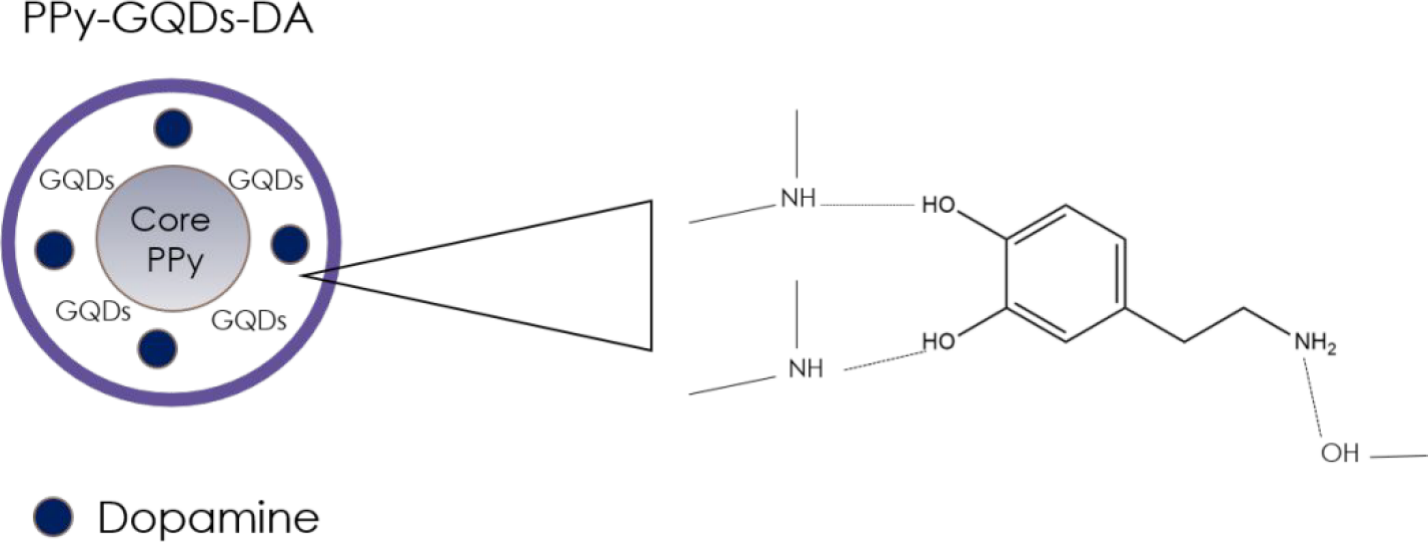
Detection of dopamine with PPy-GQDs.

A sensing process based on similar interactions between oxygen-containing groups and amino groups with DA was exploited by Zhou et al.^73^ using a sensor consisting of polypyrrole/graphene quantum dots (PPy/GQDs) core/shell hybrids (Figure 11), which showed a fluorescence emission three times greater than that of pristine GQDs. The developed sensor, contrary to that of Liu et al.,^65^ allows the determination of the target analyte based on the decrease of the fluorescence intensity that occurs with the addition of DA. The obtained device, with a LoD of 10 pM, proved to be specific for DA, and it has been efficiently tested for real sample investigations (human serum and urine samples), showing very good recovery values (97.6%–103.3%).

A new class of carbogenic nanomaterials, highly bright multicolor fluorescent sulfur-doped carbon dots (CNDs), obtained by a single-step reaction, was used for sensitive DA detection by Gupta and Nandi.^66^ The structure of these materials is still unknown, but their features of photostability, cell permeability, high brightness, and nontoxicity make them particularly suitable for use in sensing. The detection method was based on the quenching of the CNDs fluorescence at 425 nm when excited at 310 nm. The proposed materials exhibited a quantum yield up to 58%, allowing the achievement of very good performances in terms of detection limit (47 pM in water and 92 pM in blood plasma, respectively).

Metal-enhanced fluorescence based on gold nanoflowers (AuNFs) for sensitive and selective DA detection was adopted by Li et al.^59^ in 2022. In this strategy, dopamine had a double role: the analyte and a spacer between Tb(III) and AuNFs, and electrostatic interactions were mainly responsible for bonding between the Tb^3+^–DA complex and the NPs. The enhancement of the fluorescence signal of the system was possible because of the metal-enhanced fluorescence, the Tb^3+^–DA combination, and the energy transfer from the analyte to Tb^3+^. The results showed a wide linear range (0.80–300 nM), a low LOD of 0.21 nM, and satisfactory recovery in serum and DA injection samples.

A different sensing process approach based on the DA polymerization was successfully exploited in different ways by Weng et al.^9^ and by Baluta et al.^74,76^ It is, in fact, well-known that DA tends to self-polymerize to polydopamine in alkaline conditions, and the polymer possesses fluorescence capability; therefore, it can be used for optical measurements.^74^ Weng and co-workers in 2015 and Baluta and co-workers in 2017 reported rapid and easy fluorescence-sensing strategies based on the polydopamine thin film formed and absorbed on the surface of GQDs. In both cases, the detection process was due to the quenching of fluorescence which occurred through FRET. Baluta and co-workers^76^ were also responsible for the design and development of an enzyme-based fluorescent biosensor consisting of a low-temperature cofired ceramic platinum electrode (LTCC-Pt) covered with a thin film of poly(dithienotetraphenylsilane) and GQDs on which laccase had been immobilized. The idea to use laccase was derived from the fact that it catalyzes DA oxidation to dopamine-*o*-quinone, which is unstable and rapidly polymerizes in an alkaline environment to polydopamine, which is the desired species for the detection process. Detection limits between 8 and 80 nM were achieved using these strategies. These values do not allow the use of the just-described (bio)sensors in biological samples, but they are, however, suitable for the analysis of DA-containing drugs (e.g., dopamine injections).

Another biosensor, specifically a fluorescent aptasensor, was developed by Huang et al.^54^ in 2016. It is a system based on [Ru(bpy)_2_dppz]^2+^ (bpy = 2,2′-bipyridine and dppz = dipyrido[3,2-a:2′3′-c]phenazine) and thioglycolic acid (TGA)-capped CdTe quantum dots for DA, adenosine, and 17β-estradiol detection. The mechanism was based on the ionic conjugation between the Ru complex and QDs due to their electrostatic attraction in an aqueous solution that causes a decrease in the fluorescent intensity of QDs. After the addition of an adequate aptamer DNA, the fluorescence of QDs can be recovered thanks to the strong tendency of DNA to bind to the Ru complex. When the aptamer was first incubated with the target analyte, it could not bind to the Ru complex, and the fluorescence was quenched. It is a very highly selective and sensitive method that takes advantage of the aptamer specificity and the excellent fluorescence properties of CdTe-QDs. In addition, the authors declare that this method is one of the simplest and one of the first that can detect these three analytes by one universal system.

CdTe-QDs coated with silica were used as fluorescent probes by Xiangzhao et al.,^57^ who reported the development of a nanosensor for DA and glutathione detection in human serum samples with satisfactory results. The particularity of this device is that it can be considered a double sensor: (i) dopamine–quinone derived from the oxidation of DA, adsorbed on the surface of silica due to hydrogen bonds and electrostatic interactions, can be determined by the quenching of the photoluminescence of the modified CdTe-QDs, and (ii) glutathione, which is a strong reducing species, can be determined through the restoring of the fluorescence of QDs due to the reduction of dopamine–quinone.

The optimum features of modified QDs were also exploited by Mu et al.^55^ and Zhao et al.,^53^ who synthesized, as DA sensors, adenosine-capped CdSe/ZnS quantum dots (A-QDs) and (3-aminopropyl)triethoxysilane (APTES)-capped ZnO-QDs, respectively. In both cases, QDs were water-soluble, and the sensing processes were based on the fluorescence quenching of the QDs caused by an electron transfer. Mu and co-workers^55^ achieved a detection limit of 29.3 nM, high selectivity, and satisfactory recovery values (94.80%–103.40%) in human urine samples. Likewise, APTES-ZnO-QDs allowed a LOD value of 12 nM to be obtained with no significant interference effects from a wide plethora of common molecules present in human blood serum.

Zhang et al.^70^ described a novel aptamer-based biosensor for dopamine determination using fluorescence energy transfer between a nanomaterial—single-wall carbon nanohorns (SWCNHs)—and a fluorescein derivative. In the proposed strategy, an aptamer was labeled with 5-carboxyfluorescein (FAM) and, because of π–π interactions, could be absorbed on the surface of SWCNHs, which led to a decrease in fluorescence intensity. In contrast, in the presence of DA, a G-quadruplex formed when the analyte bonded to the labeled aptamer could not interact with the NM surface, resulting in the recovery of fluorescence. The platform exhibited a linear range of 0.02–2.2 mM and a detection limit of 5 μM, and its applicability was confirmed by the analysis of dopamine-spiked serum samples.

Table 7 summarizes and compares some nanomaterial-based fluorescent biosensors for DA detection.

**Table 7:** Nanomaterials-Based Fluorescent Biosensors for Dopamine Detection^a^

(bio)sensor	linear range	LOD	ref
B–N-CDs	1 pM–1 μM	0.1 pM	(65)
PPy-GQDs	5–8000 nM	10 pM	(73)
CNDs	0–20 μM	47 pM	(66)
Au nanoflowers/Tb^3+^	0.8–300 nM	0.21 nM	(59)
GQDs	0–60 μM	0.008 μM	(9)
GQDs	1–40 μM	0.022 μM	(74)
lac-polymer-GQDs	1–200 μM	80 nM	(76)
aptamer–Ru complex-QDs	0.03–0.21 μM	19 nM	(54)
CdTe-QDs@silica	0.5 μM–0.1 mM	0.241 μM	(57)
CdSe/ZnS-QDs	100 nM–20 μM	29.3 nM	(55)
APTES-capped ZnO-QDs	0.05–10 μM	12 nM	(53)
FAM-DNA/SWCNHs	0.02–2.2 mM	5 μM	(70)
N-doped carbon NPs	0.5–50 μM	140 nM	(69)

a F-CuInS_2_ = 3-aminophenyl boronic acid-functionalized CuInS_2_; ZnSa = zinc-salophen; TGA = thioglycolic acid; ds-DNA = double-stranded DNA; GSH = glutathione, ATTO-590 *N*-hydroxysuccinimidylester (NHS ester) dye.

### 5.4 Detection of Non-catecholamine Neurohormones

**Figure 12:**
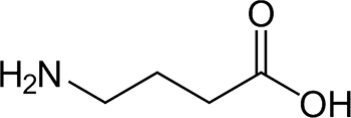
Chemical structure of γ-aminobutyric acid (GABA).

Gamma-aminobutyric acid (GABA) (Figure 12) is one of the most important inhibitory neurotransmitters in the central nervous system. Disturbances in the GABA level result in the development of neurological diseases such as epilepsy, Alzheimer’s disease, and panic disorder. As a neurotransmitter, it has special properties in the human body, for instance, the ability to lower blood pressure, improve long-term memory, and control insulin secretion.^71,172−174^ Numerous studies have been conducted to find an effective and efficient method of production of this neurotransmitter—currently, GABA is produced in pharmaceuticals and food in sprouted brown rice, anaerobically incubated tea leaves, or fermented milk products.^175,176^ Unfortunately, owing to the zwitterionic nature of GABA (the amino and carboxyl groups are adequately protonated and deprotonated), the detection of these compounds is an extremely difficult task; hence, the design of sensor devices is quite a challenge.^177^

**Figure 13:**
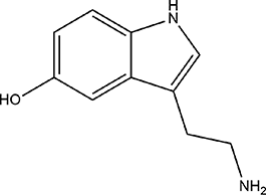
Chemical structure of serotonin.

Serotonin (Figure 13), also known as 5-hydroxytryptamine (5-HT), is a key monoamine neurotransmitter that plays crucial roles in the regulation of several behavioral and physiological functions, such as mood, sleep, appetite, anxiety, sexuality, memory, emesis, and cognition. It is also an important hormone in peripheral tissues in which it regulates a number of processes, including gastrointestinal motility, insulin secretion, vasoconstriction, and glucose metabolism.^50,75,183^ Usually, serotonin contributes to the feelings of happiness, and for this reason it is also used as a drug for depression therapy.^63,75^ However, the actual biological function of serotonin is complex and multifarious, and its low levels can be synonymous of depression, anxiety neurosis, obsessive–compulsive disorder, and migraines. Conversely, extremely high levels of serotonin could cause fatal serotonin syndrome because of its toxicity, as well as irritable bowel syndrome.^50,63^ A typical concentration of 5-HT in whole blood and in platelet-poor plasma was found at 774 ± 249 and 5.17 ± 4.17 nM, respectively,^184^ between 0.52 and 1.2 nM in cerebrospinal fluid,^185^ and in the range 10–78 μmol/mol creatinine in urine.^186^

**Figure 14:**
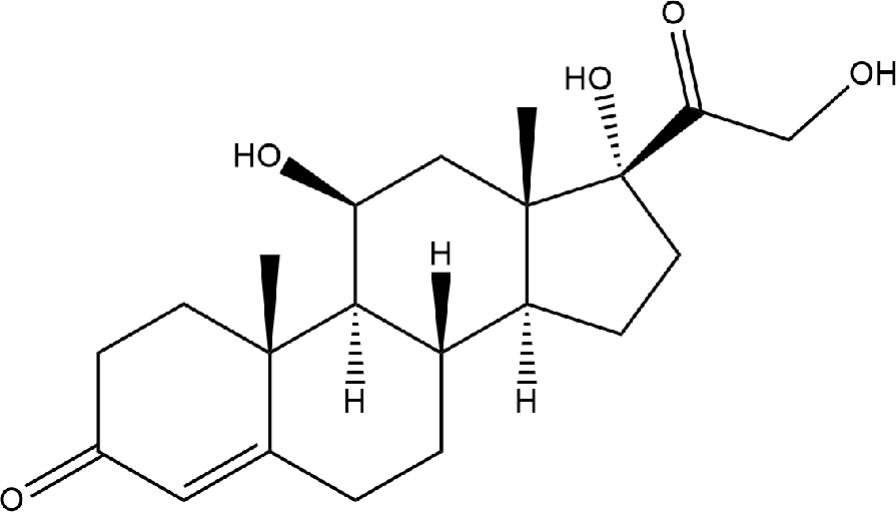
Chemical structure of cortisol.

Cortisol (Figure 14), also named hydrocortisone, is a steroid hormone that regulates various functions, such as blood pressure, glucose levels, metabolism, and immune response. It also plays a central role in the homeostasis of the cardiovascular, renal, skeletal, and endocrine systems. Cortisol secretion follows a circadian rhythm with the highest levels during the day and gradually lower at night, and they depend also on diets and physical activity. High variations of cortisol levels occur during stressed status; hence, it is considered the major stress hormone.^46,138,187^ Anomalous increments in cortisol concentration can inhibit inflammation, depress the immune system, and increase fatty and amino acid levels in blood. Extremely high levels can cause Cushing’s disease, while decreased cortisol levels lead to Addison’s disease.^187,188^ Levels of this hormone in saliva closely reflect the levels of unbound cortisol in blood; hence, saliva is the most preferred sample for analysis.^188,189^ Cortisol levels in the body fluctuate during the day between 5 nM and hundreds of nanomoles, reaching a minimum of <2 nM in the evening or at midnight.^46^

As in the case of DA, researchers are trying to develop increasingly sensitive methods for the determination of GABA, 5-HT, and cortisol. Compared to what has been observed for DA, nanomaterial-based fluorescent (bio)sensors for the detection of GABA, 5-HT, and cortisol are fewer, and the most recent are reported in the following paragraphs.

Sangubotla and Kim developed a fluorescent enzyme sensor using nontoxic carbon dots synthesized from corn juice for the sensitive and rapid detection of GABA.^71^ In order to obtain the detection platform, the functionalization of the CDs was performed with 3-aminophenylboronic acid (APBA) and nicotinamide adenine dinucleotide phosphate (NADP^+^) by means of an ECD/NHS coupling reaction. The CDs modified in this way, together with the enzyme GABase, were used for the determination of GABA by fluorescence quenching due to electron transfer between the enzyme and the substrate, thanks to the formation of a reduced form of NADPH. The system constructed in this way enabled the determination of the GABA concentration in the linear range of 0–90 μM with a detection limit of 6.46 μM. The performance of the sensor has been confirmed by testing on biological samples, such as human spinal fluid and serum.

Zhao et al. constructed a quantum dot fiber optic sensor for the direct detection of GABA by quenching and fluorescence recovery of QDs.^178^ The surface of the optical fiber was modified with QDs via an EDC/NHS coupling reaction. Then, the functionalization of the QDs was performed using 3-aminophenylboronic acid and NADP^+^. The basis of the detection was the measurement of the change in the intensity of the QDs fluorescence, which occurs due to the transfer of electrons from the QDs to NADP^+^. The use of the GABase enzyme in the system allowed the reduction of NADP^+^ to NADPH during the conversion of GABA to succinic acid. The reduction of NADP^+^ to NADPH made it difficult to transfer electrons, which made it possible to return the fluorescence intensity of the QDs to the initial state.

**Figure 15:**
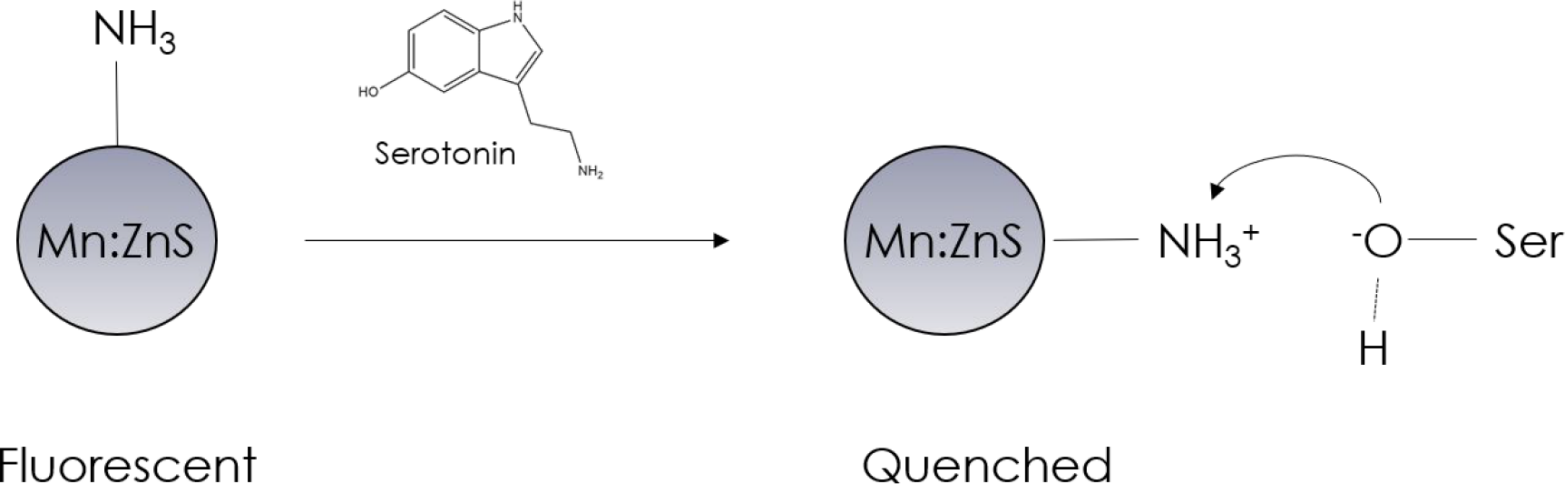
Detection of serotonin with QDs@SiO_2_@MIPs.

Mn^2+^-doped ZnS-QDs modified with silica nanoparticles based on molecularly imprinted polymers (SiO_2_@MIPs) were designed and realized by Wang et al.^50^ In such a device, advantages of QDs such as large Stokes shift, low background noise, and a narrow and symmetric emission spectrum have been combined with the peculiar feature of the MIPs to mimic a natural receptor. As a matter of fact, the sensing process was based on the formation of a complex between the amino group present in QDs@SiO_2_@MIPs and the hydroxyl group of 5-HT, which led to the quenching of the QD fluorescence (Figure 15). The excellent sensitivity and selectivity of the sensor were exploited to determine serotonin in human serum samples with recovery values between 99.71% and 100.4%, indicating that this device can be a very powerful and promising tool for clinical diagnosis application.

A supramolecular chemistry-based approach combined with the use of reduced graphene oxide (rGO) was instead employed by Zhao and co-workers^75^ very recently. They used a new class of macrocyclic species called multifarenes, which are molecules consisting of 4-*t*-butylphenol and 2-imidazolidinethione units. They realized a chemosensor with a double response in fluorescence and voltammetry, consisting of multifarenes[3,3] hybridized with rGO. In particular, fluorescence measurements were carried out in both DMSO, at 341 nm, and water, at 339 nm. In both cases, the fluorescence emission of serotonin hydrochloride solutions was quenched with the addition of multifarenes[3,3] containing sulfur atoms, denoting the formation of a host–guest complex. The obtained device with a detection limit of 0.055 μM was used for the detection of 5-HT in human serum, giving recovery values between 93.9% and 105.5%.

A device for the highly selective detection of 5-HT, based on a new class of nanomaterials, gold nanoclusters (AuNCs), was developed in 2019 by Sha and co-workers.^63^ Gold nanoclusters as well as QDs possess a large Stokes shift, good photostability, and ultrasmall size. In particular, the system developed by Sha et al. is based on the use of transferrin-encapsulated gold nanoclusters (Tf–Au-NCs) because of the well-known high affinity between sialic acid (SA) and 5-HT,^190,191^ due to the presence of SA residues in transferrin. Moreover, transferrin, with 40 cysteine and 26 tyrosine fragments in its chain, is a very suitable reducing and stabilizing agent for gold nanoclusters. The obtained biosensor is a turn-on type device, as it showed an aggregation-enhanced emission of Tf–Au-NCs in the presence of 5-HT. With a detection limit of 0.049 μM and a linear range of 0.2–50 μM, it was applied for 5-HT quantification in human serum.

Similarly to the 5-HT sensor developed by Wang et al.,^50^ the advantageous feature of the molecular imprinting technology to obtain a tailor-made specific binding cavity for a certain target molecule was also exploited by Murase and co-workers^192^ in the construction of a sensor for cortisol detection. They reported the synthesis of a fluorescence polarization assay platform based on core–shell-type molecularly imprinted polymer particles (MIP-NPs) using cortisol-21-monomethacrylate as a template agent. The sensing process was based on the competitive binding of dansyl-labeled cortisol and cortisol against the cortisol-imprinted nanocavities. The just-mentioned device exhibited a detection limit of ca. 80 nM, and it showed no effects of progesterone interference.

**Figure 16:**
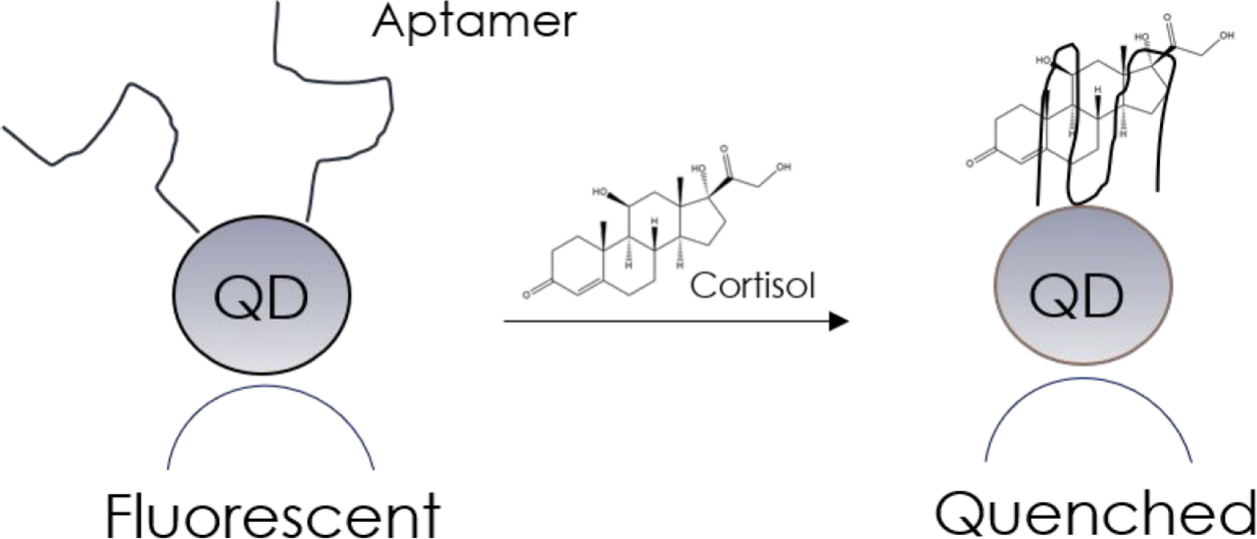
Detection of cortisol with the cortisol-selective aptamer conjugated on CdSe/ZnS-QDs.

A more recent work, published in 2020 by Liu et al.,^46^ reported instead two new fluorescence biosensors, one based on cortisol-selective aptamers conjugated on the CdSe/ZnS core–shell QDs surfaces and the other on anticortisol antibodies conjugated to the same ones. In both cases, the modified QDs were carried by magnetic nanoparticles in order to facilitate probe conjugation and cortisol detection in saliva samples. In the presence of cortisol, the authors observed fluorescence quenching of QDs (Figure 16), revealing concentrations of ca. 1 nM for the aptamer-based sensor and ca. 100 pM for that based on antibodies.

The sensor systems for the detection of GABA, serotonin, and cortisol described above are summarized in Table 8.

**Table 8:** Fluorescent Sensors for the Determination of Non-catecholamine Neurohormones

sensing platform	analyte	linear range	LOD	ref
CDs/ABPA/NADP^+^	GABA	0–90 μM	6.46 μM	(71)
QDs/ABPA/NADP^+^	GABA			(178)
QDs@SiO_2_@MIPs	5-HT	0.28–2.8 μM	3.91 nM	(50)
multifarenes[3,3] hybridized with rGO	5-HT		55 nM	(75)
Tf–Au-NCs	5-HT	0.2–50 μM	49 nM	(63)
MIP-NPs	cortisol		80 nM	(192)
Ab–CdSe/ZnS-QDs	cortisol		100 pM	(46)
aptamer–CdSe/ZnS-QDs		0.4–400 nM	1 nM	

## 6 Conclusions and Prospects

Neurotransmitters and hormones, such as epinephrine, dopamine, and cortisol, are vital for maintaining good health, both physical and mental. Even small changes in their levels in body fluids may indicate the presence of various conditions, including neurodegenerative and cardiological problems. This is why sensitive and fast methods for the determination of these biomolecules are required. This need is met by the development of optical sensors that provide a convenient way to monitor neurohormones in the human body. The incorporation of nanomaterials, such as quantum dots, carbon nanotubes, or gold nanoparticles, enables the improvement of sensing and biosensing platforms, leading to better results, faster response times, and device miniaturization. Various nanomaterials possess optical properties that can be directly applied in the detection; moreover, they can also be modified to adapt to specific needs and requirements. As presented in this Review, nanomaterial-based fluorescent sensors can be used to determine low and, more importantly, medically relevant concentrations of neurotransmitters and hormones, which leads to better prevention, diagnostics, and treatment of diseases, improving overall healthcare.

The presented Review describes neurohormones–dopamine, cortisol, epinephrine, serotonin, GABA, norepinephrine, and fluorescence-based sensing strategies using nanomaterials. In order to construct fluorescence-based sensors and biosensors for applications in medical diagnostics, multidisciplinary cooperation and overcoming a number of challenges are necessary. Prospects in the case of optical sensors or biosensors are mainly focused on miniaturization and cost-effectiveness. Below, the most important challenges facing (bio)sensorics development from a construction point of view are presented.

Apart from the most important working parameters of biosensors for diagnostic application, which are sensitivity and selectivity, devices for clinical application should allow miniaturization with simultaneous integration, automatization, and multiplex detection without the need for sophisticated apparatus and trained personnel.^193^ The most approachable in this context are microfluidic devices, which often are used in miniaturized optical bioanalytical systems. Microfluidic approaches, which can be compared to miniaturized forms of biochemical laboratories, allow the execution of many analyses at the same time, mixing or separation of reagents, biochemical reaction monitoring, and signal output.^194,195^ However, despite these advantages, as well as many available sources describing the operation of miniaturized optical sensors/biosensors, also based on microfluidic platform-assisted miniaturized biosensing systems, there is still a problem with many analysis steps and with the integration of sample pretreatment. Microfluidic systems could overcome these problems to some extent; however, the main challenge still remains—to develop a fully integrated detection system in a solid and approachable format.

Another important issue is cost-effectiveness. It is important in sensor development to use ecofriendly, nontoxic, and relatively cheap components. Solutions may be to use stable mass production that will reduce costs or to focus on inexpensive disposable chips with replaceable components that can avoid cross-contamination problems and complicated cleaning procedures when handling biological samples, i.e., integration schemes that enable disposable cartridges and stand-alone readers.^196,197^ Additionally, biosensors can be combined with smartphones as their cameras, light sources, image processing, and communication possibilities can lower costs and simplify distribution on a large scale and reduce the time of work.^198^ Such types of chips can be used to measure signals directly from patient samples, analyze data with personalized applications, and wirelessly send the results for interpretation.^199^ With the use of microfluidic-based biodevices, costs could be reduced thanks to using low-cost material and small-volume reagent requirements.

To summarize, apart from manipulations within sensitive and selective detection (i.e., new possibilities of biodetection and combinations with nanomaterials facilitating the detection process), integration, coherence, and miniaturization are the most important challenges facing biosensors in the context of their commercialization. The use of fluorescent methods for the determination of medically important neurohormones described in this Review only highlights the need to introduce such devices to the market as soon as possible, as neurohormones are indicators of the body’s homeostasis.

## Vocabulary

Sensors: detection platforms that convert chemical information into a useful signal.
Biosensors: sensors in which biomolecules, such as enzymes of nucleic acid fragments, are involved in the detection process.
Neurotransmitters: signaling molecules secreted by the body, essential for the functioning of the neural systems.
